# Dermatology education in U.S. ophthalmology residency: a survey of the program directors

**DOI:** 10.1186/s12909-024-06583-9

**Published:** 2025-01-30

**Authors:** Winnie Fan, Rojina Nekoonam, Saras Ramanathan, Amanda Twigg

**Affiliations:** 1 San Francisco School of Medicine, University of California, San Francisco, CA USA; 2 Department of Ophthalmology, University of California, San Francisco, CA USA; 3 University of California San Francisco, Department of Dermatology, 1701 Divisadero St. 3rd Floor, San Francisco, San Francisco, CA 94115 USA

**Keywords:** Ophthalmology residency education, Interdisciplinary training, Dermatology knowledge

## Abstract

**Background:**

Many ocular conditions have associated dermatological findings. However, the inclusion of dermatology education in U.S. Ophthalmology residency programs remains limited. This study aims to characterize dermatology education in U.S. ophthalmology residency programs through program directors’ (PDs’) opinions.

**Method:**

The authors designed and electronically distributed a national survey in August 2022 to PDs of 124 ophthalmology residency programs. The survey instruments examined the availability and characteristics of dermatology rotation, the PDs’ perceptions of such rotation, and their perspectives on trainees’ dermatology knowledge. Descriptive statistics were used to summarize survey responses. Sample t-tests were used to compare responses between PDs from programs with and without dermatology rotation.

**Results:**

49 PDs (39.5%) responded to the survey. Most programs (*n* = 27 [61.4%]) did not offer dermatology rotations and, of these programs, most (*n* = 15 [83.3%]) did not consider increasing dermatology exposure important. 57.1% (*n* = 8) of PDs at residency programs with dermatology rotation considered such education beneficial for their trainees. Most PDs do not consider their residents comfortable with procedures such as laser or cryotherapy, procedures relevant to ocular care.

**Conclusion:**

Dermatology rotations were uncommon among U.S. ophthalmology residencies. Perceptions towards dermatology education varied among PDs, with those from programs with dermatology rotation expressing more favorable opinions.

**Supplementary Information:**

The online version contains supplementary material available at 10.1186/s12909-024-06583-9.

## Background

Many ocular conditions have dermatological components that ophthalmologists frequently encounter [1]. Conditions such as atopic dermatitis and rosacea are often associated with ocular surface disease. Autoimmune and multisystem disorders like lupus, sarcoidosis, and Sjögren's disease commonly present with both cutaneous and ocular manifestations. Additionally, ophthalmic medications can cause periorbital skin side effects, such as hyperpigmentation from prostaglandin analogs. Dermatologic principles, such as those applied in procedures (e.g., excising periocular neoplasms), and managing wound healing, are particularly relevant to ophthalmologists. This is especially true for those specializing in oculoplastic surgery.

A strong foundation in both dermatology and ophthalmology can facilitate early recognition and multidisciplinary management of these oculocutaneous conditions, leading to better patient outcomes. For example, a prospective interventional study showed that ophthalmologists’ diagnostic accuracy for pigmented periorbital skin lesions significantly improved after a dermoscopy training course focused on these lesions [2].

Implementing dermatology education during ophthalmology residency may offer an effective way to address potential knowledge gaps that ophthalmologists have in skin and soft tissue diseases, wound healing, and dermatologic procedures. The Accreditation Council for Graduate Medical Education (ACGME) requires ophthalmology residency programs to include an integrated or joint preliminary program, which offers trainees interdisciplinary learning opportunities in relevant specialties, including dermatology, during their intern year. However, while the ACGME mandates preliminary training, it currently provides no further guidelines regarding continuing interdisciplinary education during ophthalmology residency. This highlights a need for understanding the current level of dermatology training in ophthalmology residency programs.

To our knowledge, the availability of dermatology education and the opinion of residency program directors on such an opportunity have not been previously examined. This survey study aimed to characterize dermatology education in U.S. ophthalmology residency programs through the opinions of residency program directors (PDs).

## Method

This cross-sectional survey study was approved by the University of California, San Francisco Institutional Review Board. A confidential 28-question Qualtrics survey was developed based on Kern’s Six Step Curriculum and the content listed on the ACGME ophthalmology program requirements [2, 3]. The survey was electronically delivered to PDs of the 124 ACGME-approved U.S. ophthalmology residency programs in August 2022. Repeat invites were sent to encourage participation. All respondents provided informed consent. Completion of the survey was optional. Missing data were excluded from the analysis. Categorical responses were summarized as counts and percentages. Free responses were categorized manually. The mean and standard deviation of the Likert scales were calculated. The mean scores between PDs from programs with dermatology rotation and programs without were compared using an independent sample *t*-test (*p* < 0.05). Additional information is available in the Supplement.

## Results

**Fig. 1:**
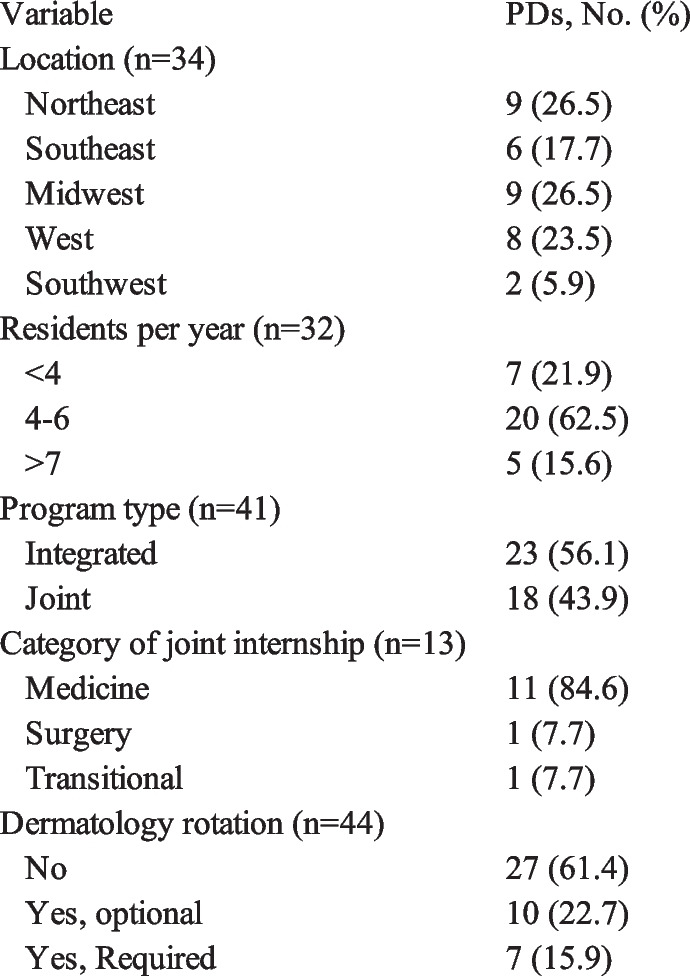
Residency program characteristics** (**PDs = program directors)

**Fig. 2:**
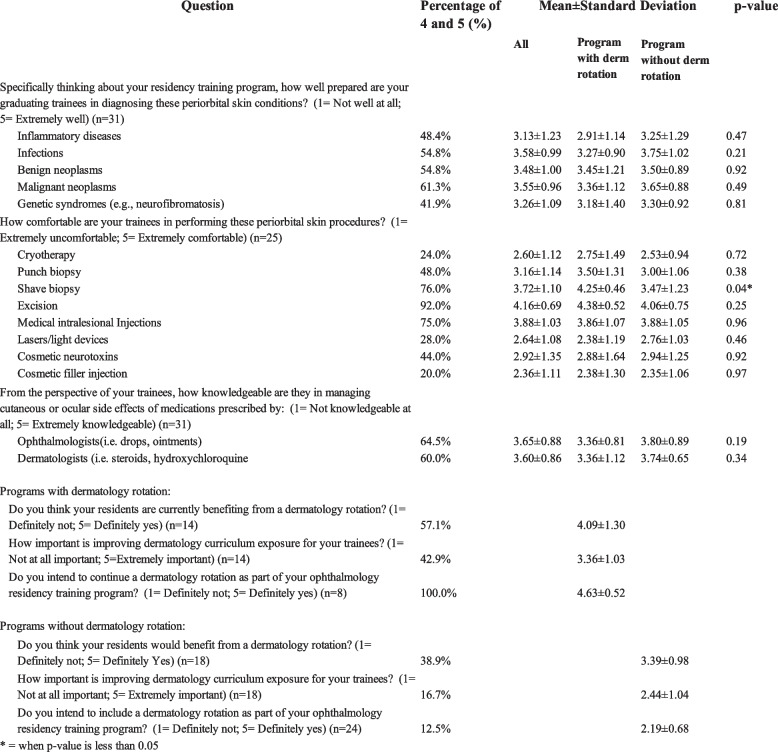
Program directors’ (PDs) perspectives on resident dermatology knowledge and dermatology rotation

Of 124 PDs contacted, 49 current PDs responded to the survey. 25.7% of the PDs were from Northeast programs, 22.8% from the South, 25.7% from the Midwest, and 22.9% from the West. Most programs (*n* = 27 [61.4%]) did not have dermatology rotations (Fig. 1), though most offered elective time (*n* = 19 [73.1%]) 2–22 weeks. PDs identified malignant skin neoplasms (*n* = 21), atopic dermatitis (*n* = 18), infections and infestations (*n* = 18), benign skin neoplasms (*n* = 18), and allergic/irritant contact dermatitis (*n* = 17) as the most relevant dermatology topics for ophthalmology training. The PDs’ perceptions of the graduating trainees’ dermatology knowledge were summarized in Fig. 2. Most PDs rated their residents as comfortable with excision, shave biopsy, and lasers, but less so with cosmetic filler injection, cryotherapy, and lasers/light devices. Notably, PDs at programs with a dermatology rotation were significantly more likely to rate their residents as comfortable with shave biopsy (*P* < 0.05).

Programs with dermatology rotation:

The duration of dermatology rotation ranged from 2–6 weeks. The most common training methods were in-person clinical experiences (*n* = 10 [45.5%]), asynchronous didactic (*n* = 5 [22.7%]), and synchronous didactic (*n* = 2 [9.1%]), with in-person clinical experiences considered the most effective (*n* = 8 [32.0%]). Most PDs (*n* = 8 [57.1%]) reported that the residents “definitely yes" or “probably” benefited from their rotation (Fig. 2). Most PDs reported no barriers to continuing the rotation (*n* = 5). Reported barriers included time constraints (*n* = 3), lack of resident interest (*n* = 1), and lack of leadership support (*n* = 1).

Programs without dermatology rotation:

Most PDs (*n* = 16 [66.7%]) stated that they would "definitely not” or “probably not" offer a dermatology rotation (Fig. 2). Obstacles to offering dermatology rotation included time constraints (*n* = 31), lack of appropriate clinic exposure (*n* = 5), and lack of resident interest (*n* = 4). In-person clinical experience was considered by most (*n* = 13 [52%]) the best learning method.

## Discussion

To the best of our knowledge, this is the first survey of dermatology education in ophthalmology residency programs. Our study found that most programs do not offer a dermatology rotation; however, among those that do, program directors considered the experience valuable for residents. This may be attributed to first-hand accounts from residents who benefited from dermatology training or to experiences at programs with greater interdisciplinary collaboration between the dermatology and ophthalmology departments.

We suspect that institutional barriers, such as limited curriculum time or the absence of a home dermatology department, may hinder the incorporation of dermatology rotations into ophthalmology residency programs. Additionally, the perception of dermatology as irrelevant to ophthalmology may contribute to a lack of emphasis on dermatology education during residency.

A previous survey of ophthalmology residents and fellows revealed that 74% of the respondents considered dermatology as relevant due to its clinical overlap with ophthalmology, yet most respondents did not have exposure to dermatology (58.6%) before residency [4]. Our survey found that most PDs perceive residents as less comfortable managing periorbital inflammatory skin diseases and performing periorbital skin procedures such as cryotherapy and laser, techniques used in treating ocular surface diseases and ocular tumors.

To encourage increased dermatology exposure during ophthalmology residency, we recommend PDs collaborate with dermatologists to develop targeted curricula and clinical experiences, such as:

- Establishing joint educational conferences or grand rounds between the ophthalmology and dermatology departments
- Incorporating dermatologic content into the ophthalmology didactic curricula
- Provide opportunities for ophthalmology residents to rotate through dermatology clinics, even if a full dedicated rotation is not feasible.

By fostering these interdepartmental collaborations, ophthalmology residency programs can better prepare their trainees to recognize, diagnose, and manage the dermatologic conditions they commonly encounter in clinical practice.

This study has several limitations. First, the 40% response rate may have introduced non-response bias, as those who responded to the survey may differ systematically from those who did not. This is further evidenced by the geographic distribution of respondents, which included a higher proportion of program directors (PDs) from the West (22.9%) than the actual distribution of ophthalmology residency programs in that region (13.5%) [5]. Second, the limited sample size may have reduced the study's power to detect statistically significant differences between groups. Finally, missing data, potentially due to survey length or low participant engagement, were excluded from the analysis. This missing data may not have been random, potentially introducing bias and affecting the results.

Future studies should address these limitations by aiming for a higher response rate, ensuring a geographically representative sample, and employing strategies to minimize missing data, such as using imputation techniques or sensitivity analyses. Future prospective studies examining comfort with dermatologic procedures and wound healing in residents who rotate through dermatology versus those who do not could provide further insight into the necessity of such interdisciplinary training.

## Conclusions

Dermatology exposure was not common among U.S. ophthalmology residencies. PDs’ perception towards dermatology education varied, with a greater portion of PDs from programs with dermatology rotation holding favorable opinions.

## Supplementary Information

Supplementary Material 1.

## Data Availability

The datasets used and/or analyzed during the current study are available from the corresponding author on reasonable request.

## Abbreviations

ACGME: Accreditation Council for Graduate Medical Education
PD: Program Director
UCSF: University of California, San Francisco
