# Supplementary material for: Dermatology education in U.S. ophthalmology residency: a survey of the program directors
Source: BMC Med Educ. 2025 Jan 30;25:151. doi: 10.1186/s12909-024-06583-9 (PMC11781010; doi:10.1186/s12909-024-06583-9)
Supplement: Supplementary file 1 — Supplementary Material 1. [file 12909_2024_6583_MOESM1_ESM.docx]

**eFigure** Study flow diagram

Program directors (PDs) to whom surveys were mailed electronically (n=124)

PDs who responded to the survey (n=54)

Excluded: Respondents were not current PDs (n=5)

Analytical sample (n=49)

**Methods** Study Methods and Survey Instruments

**Study design**

This is an IRB-approved qualitative survey study of residency PDs from ACGME-accredited Ophthalmology programs in the United States. The PDs of 124 ACGME-accredited Ophthalmology programs were identified. The PDs’ e-mail contacts were collected from these programs. The survey was conducted in Qualtrics. Consent information was provided to potential participants as the first survey question. The second question inquire how many years the participant has been a PD. If the participant selects that they are not a current PD, the survey ends and they are excluded from the study. Consent was required before accessing the rest of the survey. The survey was completely anonymous and voluntary. Respondents were not required to answer all questions. Participants did not receive monetary compensation**.**

The survey instruments were developed by the study team, which consisted of UCSF ophthalmology and dermatology residency educators, based on their teaching experiences and literature review. The survey instruments were developed based on Kern's Six-Step Curriculum Development and the content listed by the American Academy of Ophthalmology^1,2^. The 28 survey instruments included multiple-choice, free-response, and 5-point Likert scale (See **Survey Instrument** below).

The survey assessed the characteristics of the residency programs and of the residency PDs. It also inquired about the availability and characteristics of dermatology rotations and dermatology education in ophthalmology residency programs. 5-point Likert scales were used to evaluate the PDs’ opinions on a series of statements. Specifically, the respondents were asked to rate the graduating residents’ extent of (1) preparation with diagnosing various common periorbital skin conditions, (2) comfort in performing various medical and cosmetic procedures of the periorbital skin, and (3) knowledge of managing ocular or cutaneous side effects of medications prescribed by ophthalmologists and dermatologists. They were also asked to select curricular components of a dermatology rotation that would lead to success in ophthalmology.

For programs with dermatology rotations, the opinions of the PDs on the benefit and importance of such a rotation for residents were assessed. They were also asked to identify threats and challenges with continuing to offer the dermatology rotation. For programs without dermatology rotations, the opinions of the PDs on the necessity or priority of creating such a rotation were assessed. They were also asked to identify obstacles to starting a dermatology rotation.

**Data collection**

A personalized invitation, a cover letter describing the study goals, and a web link to the online survey were emailed to all residency PDs. The initial survey was distributed in August 2022, followed by a three-week reminder email. The survey was closed after four weeks.

**Statistical analysis**

Data were stored and analyzed in Microsoft Excel. Missing data were excluded from the analysis. Categorical responses were summarized as counts and percentages. Free responses were categorized manually. The mean and standard deviation of the Likert scales were calculated. The mean scores of the Likert scales were compared between PDs from programs with dermatology rotation and PDs from programs without dermatology rotation using an independent two-tailed t-test. The significance level was α = 0.05. Variables with p values < 0.05 were considered statistically significant.

**Survey instrument**

Welcome to the research study!
We are asking you to take part in a research study being done by Dr. Amanda Twigg with the Dermatology and Ophthalmology Departments at the University of California, San Francisco. Your participation in this research is **voluntary**.

 If you choose to be in the study, you will complete a questionnaire. This questionnaire will help us learn more about the current state of dermatology training in ophthalmology residency programs across the United States and perform a needs assessment for the development of such a curriculum.

 The questionnaire will take less than **5 minutes** to complete. You can skip questions that you do not want to answer or stop the questionnaire at any time.

 **Questions**? Please contact Dr. Amanda Twigg at **amanda.twigg@ucsf.edu**.
 If you have questions or concerns about your rights as a research participant, you can call the UCSF Institutional Review Board at 415-476-1814.

- I consent, begin the study
- I do not consent, I do not wish to participate

How many years have you been a program director?

- <3 years
- 3-7 years
- >7 years
- I am not a program director

How many years have you been in practice?

- 5-10 years
- 10-15 years
- 15-20 years
- >20 years

Where is your residency program located?

- Northeast
- Southeast
- Midwest
- West
- Southwest

How many residents are enrolled in your program each year?

________________________________________________________________

Select the option that best fits your residency program.

- Integrated Internship
- Joint Internship

Choose the category that most accurately describes the joint internship.

- Medicine
- Surgery
- Transitional
- Other, specify ________________________________________________

How much elective time do your residents get and during which years of training?

________________________________________________________________

Do your residents currently participate in a dermatology rotation?

- No
- Yes (optional)
- Yes (required rotation)

For programs with dermatology rotation:

How much time do your residents spend in the dermatology rotation (hours per week, number of weeks)?

________________________________________________________________

What is the nature of dermatology exposure your residents receive during residency? Select all that apply.

- Synchronous didactic curriculum (live lectures)
- Asynchronous didactic curriculum (self-paced modules)
- Industry-sponsored courses
- In-person clinical experience
- Readings (texts, journal articles, etc)
- Other conferences
- Other, specify ________________________________________________

Which format do you think would be the most effective in preparing residents for this interdisciplinary learning? Select all that apply.

- Synchronous didactic curriculum (live lectures)
- Asynchronous didactic curriculum (self-paced modules)
- Industry sponsored courses
- In-person clinical experience
- Readings (texts, journal articles, etc)
- Other conferences
- Other, specify ________________________________________________

Do you think your residents are currently benefiting from a dermatology rotation?

- Definitely not
- Probably not
- Might or might not
- Probably yes
- Definitely yes

How important is improving dermatology curriculum exposure for your trainees?

- Not at all important
- Slightly important
- Moderately important
- Very important
- Extremely important

Do you intend to continue a dermatology rotation as part of your ophthalmology residency training program?

- Definitely not
- Probably not
- Might or might not
- Probably yes
- Definitely yes

What are current threats/challenges to your dermatology rotation? Select all that apply.

- Lack of appropriate clinic exposure for ophthalmology learners
- Time constraints with other competing rotations
- Lack of resident interest
- Lack of ophthalmology leadership support, explain ________________________________________________
- Lack of dermatology leadership support, explain ________________________________________________
- None identified
- Other, please specify ________________________________________________

What additional assistance/resources would you want to support your current dermatology rotation?

________________________________________________________________

For all programs:

Specifically thinking about your residency training program, how well prepared are your graduating trainees in diagnosing these periorbital skin conditions?

|  | Not well at all | Slightly well | Moderately well | Very well | Extremely well |
| --- | --- | --- | --- | --- | --- |
| Inflammatory diseases |  |  |  |  |  |
| Infections |  |  |  |  |  |
| Benign neoplasms |  |  |  |  |  |
| Malignant neoplasms |  |  |  |  |  |
| Genetic syndromes (i.e. Neurofibromatosis) |  |  |  |  |  |

How comfortable are your trainees in performing these periorbital skin procedures?

|  | Extremely uncomfortable | Somewhat uncomfortable | Neither comfortable nor uncomfortable | Somewhat comfortable | Extremely comfortable |
| --- | --- | --- | --- | --- | --- |
| Cryotherapy |  |  |  |  |  |
| Punch biopsy |  |  |  |  |  |
| Shave biopsy |  |  |  |  |  |
| Excision |  |  |  |  |  |
| Medical intralesional injections |  |  |  |  |  |
| Lasers/light devices |  |  |  |  |  |
| Cosmetic neurotoxins |  |  |  |  |  |
| Cosmetic filler injection |  |  |  |  |  |

From the perspective of your trainees, how knowledgeable are they in managing cutaneous or ocular side effects of medications prescribed by:

|  | Not knowledgeable at all | Slightly knowledgeable | Moderately knowledgeable | Very knowledgeable | Extremely knowledgeable |
| --- | --- | --- | --- | --- | --- |
| Ophthalmologists(i.e. drops, ointments) |  |  |  |  |  |
| Dermatologists (i.e. steroids, hydroxychloroquine) |  |  |  |  |  |

What are the curricular components of a dermatology rotation that would lead to success in ophthalmology? Select all that apply.

- Atopic dermatitis
- Allergic/irritant contact dermatitis
- Immunobullous disease
- Rosacea
- Urticaria
- Other inflammatory dermatoses
- Drug reactions/side effects
- Pigmentary disorders (vitiligo)
- Benign neoplasms
- Malignant neoplasms (i.e. BCC, SCC, melanoma, sebaceous carcinoma)
- Infections and infestations
- Genetic syndromes
- Cryotherapy
- Punch biopsy
- Shave biopsy
- Excisions
- Medical intralesional injections
- Lasers/ light devices
- Cosmetic neurotoxin injections
- Cosmetic filler injections
- Mohs surgery
- Dermatopathology

Do you have any other comments or feedback that you would like to share with us?

________________________________________________________________

For programs without a dermatology rotation:

Start of Block: No

Do you think your residents would benefit from a dermatology rotation?

- Definitely not
- Probably not
- Might or might not
- Probably yes
- Definitely yes

How important is improving dermatology curriculum exposure for your trainees?

- Not at all important
- Slightly important
- Moderately important
- Very important
- Extremely important

What are some of the obstacles to having a dermatology rotation at your institution? Select all that apply.

- No dermatology program at institution
- Lack of appropriate clinic exposure for ophthalmology learners
- Time constraints with other competing rotations
- Lack of resident interest
- Lack of ophthalmology leadership support, explain ________________________________________________
- Lack of dermatology leadership support, explain ________________________________________________
- None identified
- Other, please specify ________________________________________________

Do you intend to include a dermatology rotation as part of your ophthalmology residency training program?

- Definitely not
- Probably not
- Might or might not
- Probably yes
- Definitely yes

Which format do you think would be the most effective in preparing residents for this interdisciplinary learning? Select all that apply.

- Synchronous didactic curriculum (live lectures)
- Asynchronous didactic curriculum (self-paced modules)
- Industry sponsored courses
- In-person clinical experience
- Readings (texts, journal articles, etc)
- Other conferences
- Other, specify ________________________________________________

What additional assistance/resources would you require if your institution offered a dermatology rotation?

________________________________________________________________

References

1. ACGME. ACGME Program Requirements for Graduate Medical Education in Ophthalmology. Published online June 12, 2022. https://www.acgme.org/globalassets/pfassets/programrequirements/240_ophthalmology_2023.pdf

2. Thomas PA, Kern DE, Hughes MT, Tackett SA, Chen BY, eds. *Curriculum Development for Medical Education: A Six-Step Approach*. Fourth edition. Johns Hopkins University Press; 2022.
